# Site-specific and dose-dependent effects of glucocorticoid receptor phosphorylation in yeast *Saccharomyces cerevisiae*

**DOI:** 10.1016/j.steroids.2010.03.001

**Published:** 2010-06

**Authors:** Natasa Popovic, Sabera Ruzdijic, Dusan T. Kanazir, Ana Niciforovic, Miroslav Adzic, Elissavet Paraskevopoulou, Constantia Pantelidou, Marija Radojcic, Constantinos Demonacos, Marija Krstic-Demonacos

**Affiliations:** a VINČA Institute of Nuclear Sciences, P.O. Box 522, 11001 Belgrade, Serbia; b Institute for Biological Research ‘Sinisa Stankovic’, Belgrade, Serbia; c Serbian Academy of Sciences and Arts, Belgrade, Serbia; d Faculty of Life Sciences, University of Manchester, Manchester, United Kingdom; e School of Pharmacy, University of Manchester, Manchester, United Kingdom

**Keywords:** Transcription, Glucocorticoid receptor, Phosphorylation, Yeast

## Abstract

The glucocorticoid receptor (GR) signal transduction and transcriptional regulation are efficiently recapitulated when GR is expressed in *Saccharomyces cerevisiae*. In this report we demonstrate that the *in vivo* GR phosphorylation pattern, hormone dependency and interdependency of phosphorylation events were similar in yeast and mammalian cells. GR phosphorylation at S246 exhibited inhibitory effect on S224 and S232 phosphorylation, suggesting the conservation of molecular mechanisms that control this interdependence between yeast and mammalian cells.

To assess the effects of GR phosphorylation the mutated GR derivatives T171A, S224A, S232A, S246A were overexpressed and their transcriptional activity was analysed. These receptor derivatives displayed significant hormone inducible transcription when overexpressed in *S. cerevisiae*. We have established an inducible methionine expression system, which allows the close regulation of the receptor protein levels to analyse the dependence of GR function on its phosphorylation and protein abundance. Using this system we observed that GR S246A mutation increased its activity across all of the GR concentrations tested. The activity of the S224A and S246A mutants was mostly independent of GR protein levels, whereas the WT, T171A and S232A mediated transcription diminished with declining GR protein levels. Our results suggest that GR phosphorylation at specific residues affects its transcriptional functions in a site selective manner and these effects were directly linked to GR dosage.

## 1 Introduction

Glucocorticoid receptor (GR) is a member of the nuclear hormone receptor superfamily of transcription factors. GR is the intracellular, ligand-regulated transcription factor that plays an important role in numerous cellular processes and responses to extracellular signals [1]. GR is ubiquitously expressed in mammalian cells and in the absence of ligand is complexed with chaperones such as heat shock protein 90. Once bound to the ligand, GR is activated, dissociates from chaperones and translocates into the nucleus, where it binds to glucocorticoid responsive elements (GREs) and activates or represses the transcription of numerous target genes depending on cell type, promoter context and physiological settings [1]. Multiple factors such as interaction with the chaperone complexes, binding affinity and type of GREs, intracellular trafficking, interaction with numerous co-activators and co-repressors and covalent modifications determine GR transcriptional activity [1–3].

One of the most important and extensively studied post-translational modifications of GR is phosphorylation. Seven phosphorylation sites have been identified in GR isolated from mouse and hamster cells [4]. Glucocorticoid receptor expressed in yeast (*Saccharomyces cerevisiae*) is phosphorylated at T171, S224, S232, and S246 [5]. GR phosphorylation at these sites located within the N-terminal transactivation domain was reported to regulate various functional properties of this transcription factor [6,7]. For example, phosphorylation was suggested to control receptor stability and nuclear localization and to facilitate GR sumoylation [8–10]. However, GR phosphorylation has been shown to exert stimulatory and inhibitory effects on its transcriptional functions in a target gene specific manner [5,10,11]. The N-terminal transactivation domain of GR is a target for several signalling pathways and kinase cascades. Mitogen activated protein kinases (MAPKs) phosphorylate the rat GR at T171 and S246, whereas cyclin dependent protein kinases (CDKs) phosphorylate S224 and S232 [5]. The T171 site is targeted by glycogen synthase kinase-3 (GSK-3) dependent phosphorylation [5,7]. The JNK subfamily of the MAP kinases phosphorylates GR modifying most efficiently the S246 residue [10,12]. Reduced receptor-dependent transcriptional enhancement in yeast strains deficient in the catalytic or regulatory subunits of CDKs has been reported. On the contrary, deletions of FUS3 and KSS1 MAP kinase homologues in yeast increase GR mediated transcription suggesting that GR phosphorylation by these kinases may have inhibitory effect on its function [5].

Although several studies have provided insight into the effects of phosphorylation on GR function, numerous mechanistic details of this control are still not known. It has previously been shown that GR phosphorylation pattern and transcriptional activity can efficiently be recapitulated in yeast *S. cerevisiae* [5,13,14]. In yeast ectopically expressing GR, phosphorylation occurs rapidly and is observed in both the absence and the presence of hormone treatment. Phosphorylation of T171 and S246 occurred in both the absence and the presence of hormone and S224 and S232 phosphorylation increased after the addition of hormone to the yeast media. GR phosphorylation studies in mammalian cells have suggested that the modification of one site affects phosphorylation and/or other post-translational modifications of residues in the vicinity. For example, phosphorylation of GR at S224 inhibits S246 phosphorylation or vice versa, whereas the consequence of the JNK pathway activation and hence S246 phosphorylation is the augmentation of GR sumoylation [5,10,15]. Despite above mentioned and other studies [16,17] indicating the importance of phosphorylation for the function of a plethora of transcription factors there are very few reports describing effects of this modification on protein–protein contacts for GR [18]. The complexity of receptor's phosphorylation and difficulty of monitoring the relatively subtle effects of phosphorylation site mutations on protein function prompted us to explore the possibility of developing a more tractable system to approach this question. Given the similarity of the events mediated by the phosphorylated GR in yeast with those taking place in mammalian cells together with the simplicity and genetic manipulability of the yeast we have chosen *S. cerevisiae* to study the phosphorylation dependent GR activities and have established expression system where GR protein concentration can be carefully regulated.

In the present report, based on the analysis of phosphopeptide patterns of the wild type and mutant receptor, we verify that hormone dependency and interdependency of phosphorylation sites observed in mammalian cells is preserved in yeast. Furthermore, we provide evidence that the effects of the receptor phosphorylation are residue specific and closely linked to the quantity of the receptor protein in the cell.

## 2 Experimental

### 2.1 Yeast and mammalian cells

The triple protease deficient yeast *S. cerevisiae* strain, BJ2168 (a, pep 4-3, prc 1–417, prb 5–1122, ura 3–52, trp 1, leu 2) [19] was used, and the expression and reporter vectors were introduced as described below. Yeast cultures were propagated at 30 °C in minimal yeast medium with amino acids and 2% glucose. Transformations were performed by the lithium acetate procedure [20]. GRH2 rat hepatoma cells have integrated copies of the rat GR cDNA and express increased levels of the glucocorticoid receptor [21].

### 2.2 Plasmids

The yeast expression plasmid pG-N795 [13] carries the rat glucocorticoid receptor [GR] cDNA expressed from the yeast glyceraldehyde-3-phosphate dehydrogenase [GPD] promoter. This plasmid is a 2μ vector (10–40 copies per cell) with the TRP1 selectable marker. Reporter plasmid pΔs26x contains three tandem 26 bp oligonucleotides from the tyrosine aminotransferase, URA3 selectable marker and has been described previously [5,22]. Expression plasmid p414 Met25 has previously been described [23] and is a gift from M. Funk. The rat glucocorticoid receptor cDNA WT, and mutant derivatives T171A, S224A, S232A and S246A were obtained by isolating fragments carrying rat GR cDNA from the PGN795 plasmid and inserting them into the BamH1 site of p414 MET25 vector. All constructs were confirmed by restriction digestion and sequencing.

### 2.3 Metabolic labeling and phosphopeptide mapping

Metabolic labeling and the phosphopeptide mapping experiments were performed as described before [14]. Briefly, the yeast strain BJ2168 containing the GR expression vector pG-N795 was grown to O.D. 600 nm 0.4–0.7 in 50 ml of minimal selective yeast medium with amino acids and 2% glucose. The cells were washed once and incubated for 30 min at 30 °C in 50 ml phosphate-free medium. The cells were labeled with (^32^P) orthophosphate (25 mCi/ml, carrier free, New England Nuclear, USA) to a final concentration of 1 mCi/ml; one portion was brought to 10 μM deoxycorticosterone (DOC) (Sigma, USA). After 2 h at 30 °C, cells were harvested by centrifugation, washed in 5 ml of cold PBS and resuspended in 350 μl of high salt lysis buffer (45 mM HEPES pH 7.5, with 10% glycerol, 1 mM Na_2_EDTA, 400 mM NaCl, 2 mM DTT, 0.5% NP40, 25 mM sodium fluoride, 20 mM β-glycerophosphate, 5 mM sodium pyrophosphate and a protease inhibitor cocktail containing 1 μg/ml each of aprotinin, leupeptin and pepstatin A, and 1 mM PMSF). An equal volume of acid washed glass beads was added and cells were vortexed for 15 min using a horizontal bead beater (Eppendorf, USA). Cell lysates were cleared by centrifugation at 12,000 × *g* for 10 min at 4 °C. The supernatant was used for the immunoprecipitation of the receptor and samples were analysed by SDS-PAGE. Polyacrylamide gels containing the labeled receptor were washed in water and dried between cellophane sheets. Following autoradiography, the GR band was excised and gel was rehydrated and eluted in 50 mM ammonium acetate, 1 mM DTT. For digestion with V8 protease, the rehydrated gel slice was placed into a microfuge tube at room temperature in 50 mM ammonium acetate, 1 mM DTT and 50 μg/ml of V8 protease (Endoproteinase Glu-C, Boehringer Mannheim), adjusted to pH 4 and incubated at 37 °C overnight. Samples were centrifuged for 5 min at 12,000 × *g* and the supernatant containing the digested peptides was evaporated to dryness in a Speedvac (Savant, Farmingdale, USA). Peptides were resuspended in 500 μl of water, dried and washed once more. Finally, peptides were dissolved in 10 μl of 15% acetic acid, 5% formic acid. Sample containing >1000 cpm was used for 2-D phosphopeptides analysis. Peptides were electrophoresed in 15% acetic acid, 5% formic acid on cellulose plates (microcrystalline cellulose adsorbent without fluorescent indicator; Kodak, USA) at 1000 V for 50 min. Plates were then dried and subjected to ascending chromatography in the second dimension for 3 h with 37.5% butanol, 25% pyridine, 7.5% acetic acid, air dried and exposed to film [24].

### 2.4 Preparation of cell extracts, immunoprecipitation and immunoblotting

The yeast strain BJ2168 containing the GR expression vector pG-N795 and vector p414 MET25N795 were grown to O.D. 600 nm 0.4–0.7 in 50 ml minimal selective yeast medium with amino acids and 2% glucose. One portion was treated with 10 μM deoxycorticosterone (Sigma, USA) for 2 h at 30 °C. The cells were harvested by centrifugation, washed once in 5 ml of cold PBS and resuspended in 350 μl of high salt lysis buffer as described above. An equal volume of acid washed glass beads was added and cells were vortexed for 15 min using a horizontal bead beater (Eppendorf). Cell lysates were cleared by centrifugation at 12,000 × *g* for 10 min at 4 °C. The supernatant was placed in a fresh tube. To immunoprecipitate the GR, an equal volume of RIPA buffer (10 mM HEPES pH 7.5, 150 mM NaCl, 0.1% Na_2_deoxycholate, 1% Triton X-100) was added to cleared cell lysates, together with 5 ml of ascities fluid containing the rat GR specific monoclonal antibody BUGR2 [25]. After 1–2 h at 4 °C, 100 μl of 50 mg/ml protein A–sepharose (Sigma, USA) equilibrated in RIPA buffer was added and incubated for additional 2 h with gentle agitation at 4 °C. Protein A–sepharose was collected by centrifugation, washed 4 times with ice cold RIPA buffer, and once with PBS. Bound protein was released in 2× SDS sample buffer (0.125 M Tris pH 6.8, 20% glycerol, 4% SDS, 10% β-mercaptoethanol and 0.004% bromophenol blue), displayed on 7.5% SDS polyacrylamide gels and transferred to Immobilon-P membranes (Millipore, USA). Immunoblots were probed with the indicated antibodies and developed with the enhanced chemiluminescence substrate according to manufacturer's instructions (Pierce Chemical Co., Rockford, IL). Intensity of the bands was quantified using Image J software.

### 2.5 Synthetic peptides

Phosphopeptides were synthesized on HMP resin using standard HOBT active ester/FMOC chemistry on an ABI 431-A peptide synthesizer (Applied Biosystems). Residues to be phosphorylated were coupled as alcohol-unprotected FMOC derivatives and were phosphorylated after synthesis with di-*t*-butyl-*N*,*N*-diisopropylphosphoramidite/tetrazole followed by the oxidation of trivalent phosphorus with *t*-butylhydroperoxide. Peptides were cleaved and deprotected with Reagent K, purified by reversed-phase HPLC and characterized by electrospray-ionization mass spectrometry (Hewlett-Packard model 5989A). 50 μg of each peptide was dissolved in 10 μl of electrophoresis buffer, loaded on TLC plates and electrophoresis and chromatography were performed as described above. Plates were dried and synthetic peptides were visualized with ninhydrin. These peptides were used as markers to identify phosphopeptides labeled *in vivo* by co-migration.

### 2.6 β-Galactosidase assay in yeast

β-Galactosidase activity was assayed as previously described [13]. Briefly, yeast cells carrying indicated GR expression vectors and pΔs26x reporter vector were collected by centrifugation from 1.5 ml of cultures and washed with Lac Z buffer (100 mM sodium phosphate buffer pH 7.0, 10 mM KCl, 1 mM MgSO_4_ and 50 mM β-mercaptoethanol). After recentrifugation, cells were resuspended in 50 μl of Lac Z buffer and permeabilized with 50 μl of chloroform and 20 μl of 0.1% SDS; β-galactosidase substrate (o-nitro-phenyl-β-galactoside, 0.7 ml of 2 mg/ml) was added and the reaction was stopped with 0.5 ml of 1 M Na_2_CO_3_ after 1–10 min incubation. The β-galactosidase activity was assayed spectrophotometricaly by measuring O.D. 420 nm. Results are presented as mean ± SEM (*n* = 6) obtained from six or more measurements. For establishing significant differences data were analysed by *t*-test and the values were considered statistically significant if the *p* value was less than 0.05.

## 3 Results

### 3.1 GR phosphorylation sites and their hormone dependency is similar in yeast and mammalian cells

**Fig. 1:**
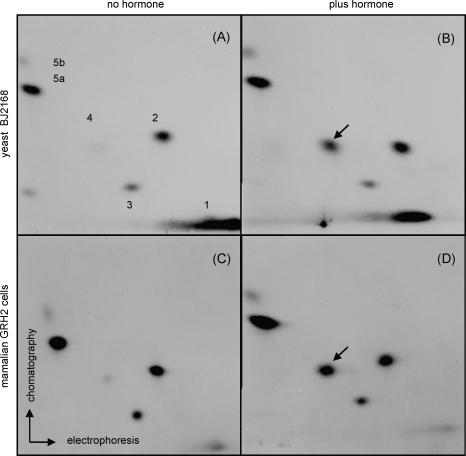
V8 phosphopeptides of glucocorticoid receptor expressed in yeast and mammalian cells. Yeast (A and B) or mammalian (C and D) cells were incubated with (^32^P) orthophosphate in the presence (B and D) or absence (A and C) of 10 μM deoxycorticosterone (DOC) and 0.1 μM dexamethasone (DEX), respectively, receptor was immunoprecipitated, resolved by SDS-PAGE, isolated from the gel and digested with V8 protease. Two-dimensional peptide mapping was performed by electrophoresis (first dimension) and chromatography (second dimension), and exposed to film and yeast pattern was compared to the known phosphopeptide pattern from mammalian cells [5]. Arrow identifies peptide whose labeling is hormone dependent.

**Fig. 2:**
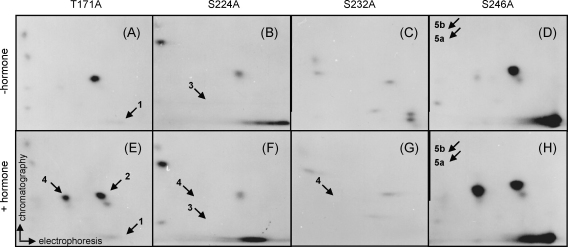
Phosphopeptide mapping of the glucocorticoid receptor phosphorylation site point mutations. Receptor mutants T171A (panels A, E), S224A (panels B, F), S232A (panels C, G) and S246A (panels D, H) were isolated from (^32^P) labeled yeast cells and cleaved with V8 proteases as described in Section 2. Phosphopeptide maps are shown for cells incubated in the absence (panels A–D) and presence (panels E–H) of hormone. Arrows identify positions of labeled peptides present in wild type receptor and lacking in mutant GR derivatives.

Phosphopeptide mapping was performed to determine the phosphorylation status of GR expressed in yeast and mammalian cells. Hormone-treated yeast and rat hepatoma cells expressing the receptor were metabolically labeled with (^32^P) orthophosphate, and the receptor was isolated by immunoprecipitation and digested with V8 protease as previously described [5]. The resulting phosphopeptides were separated on thin-layer plates in two dimensions by electrophoresis and chromatography, respectively [24]. Separate labeling reactions were carried out on hormone-treated (Fig. 1B and D) and control cultures (Fig. 1A and C). The phosphopeptide patterns of receptor labeled in yeast and mammalian cells were strikingly similar, with five major phosphopeptides generated upon V8 cleavage (Fig. 1, compare panels A and B with panels C and D). The extent of phosphorylation of these peptides seemed roughly uniform between species, except for peptide 1, which appeared more intense in yeast than in mammalian cells; in fact peptide 1 may represent a cluster of more than one peptide (Fig. 1; see also Fig. 2) whose sites are phosphorylated at low relative stoichiometries in the mammalian receptor.

### 3.2 Interdependence of GR phosphorylation sites is conserved in yeast

Earlier reports have identified seven GR phosphorylated residues in hormone-treated mouse cells, displaying different GR phosphorylation efficiencies [4]. To investigate the GR phosphorylation state we mutated four predominant sites at the corresponding rat receptor residues from serine or threonine to alanine (T171A, S224A, S232A and S246A), and characterized these derivatives in metabolically labeled yeast. V8 phosphopeptide analysis of these point mutants in hormone-treated and control cultures is shown in Fig. 2. The absence of peptide 1 is evident in the T171A mutant in both the absence and the presence of hormone (Fig. 2A and E); the residual labeling at this position is possibly due to one or more minor peptides co-migrating at that position. In a similar manner, the S246A receptor lacked peptides 5a and 5b independently of hormone treatment (Fig. 2D and H). A significant increase in the intensity of the peptides 2 and 4 in this mutant was detected potentially due to interdependency of phosphorylation sites. Notably the weakly phosphorylated peptide 3 in these experiments was evidently detected after longer exposures of all mutated derivatives apart from the S224A. The phosphopeptide pattern of the S224A mutant differed from that of the wild type receptor since the peptide 3 was absent in non-treated cells, and hormone treatment resulted in disappearance of both peptides 3 and 4 (Fig. 2B and F), whereas peptide 5a intensity was significantly increased. The S232A receptor derivative lacked the peptide 4, suggesting that S232 phosphorylation is stimulated by hormone (Fig. 2C and G). The simplest interpretation of these findings is that the peptide 4 carries hormone-dependent phosphorylation on both S224 and S232.

In fact, phosphotryptic peptide mapping of the mutant receptor derivatives in both yeast and mammalian cells supports the notion that T171 and S246 are phosphorylated constitutively, whereas the phosphorylation of S224 and S232 increases upon the addition of hormone [5,14]. Thus, each of the four receptor derivatives bearing an Ala substitution in a putative phosphorylation site lacked one or two phosphopeptides relative to the wild type receptor.

**Fig. 3:**
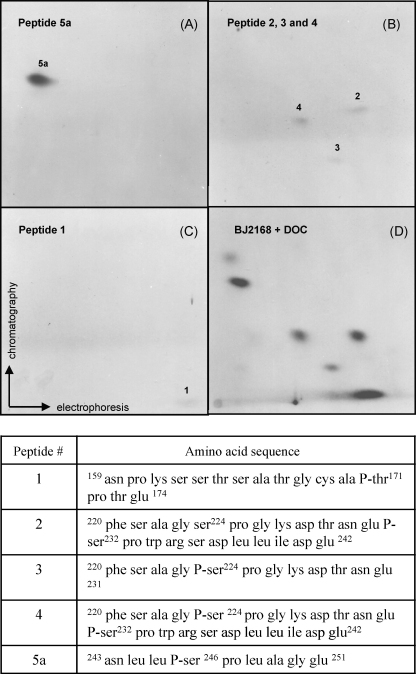
Identification of V8 peptides of the receptor and respective synthetic peptides. Individual peptides (50 μg) or subsets of peptides were fractionated by electrophoresis and chromatography, and visualized with ninhydrin. Panel A: peptide 5 carrying residue S246; Panel B: peptides 2–4, fractionated together (in other experiments, they were fractionated separately to allow their individual identification); Panel C: peptide 1; Panel D: receptor specific V8 phosphopeptides from *in vivo* labeled hormone-treated yeast cells. The sequences of respective peptides are given below.

The alteration of two peptides after the mutation of one putative target residue might be a result of partial proteolysis, or interdependence of one phosphorylation event upon another. To resolve these and other possibilities, we synthesized the predicted V8 phosphopeptides of the receptor (Fig. 3A), purified them, established their identities by mass spectroscopy, and compared their positions on 2D peptide maps with those of the receptor phosphopeptides observed after metabolic labeling. The results confirmed that phosphopeptide 5a contains a phosphoserine at position 246 (Fig. 3A), and that phosphopeptide 1 includes the phosphorylated T171 residue (Fig. 3C). The 2 and 3 peptides correspond to the 220–242 or 220–231 fragments, respectively, with a single phosphorylation site at S232 or S224, whereas peptide 4 coincides with the doubly phosphorylated version of this peptide (Fig. 3B). An interdependence of hormone-stimulated phosphorylation of these two residues may account for the intensity of this phosphopeptide and it is not due to different solubility of the peptides isolated *in vivo.* Together, these experiments produced an internally consistent interpretation of the specific residues phosphorylated in yeast cells *in vivo*, and confirmed their identification obtained *in vitro* (Figs. 1 and 3).

### 3.3 Effects of phosphorylation site mutations on receptor activity—constitutive overexpression system

**Fig. 4:**
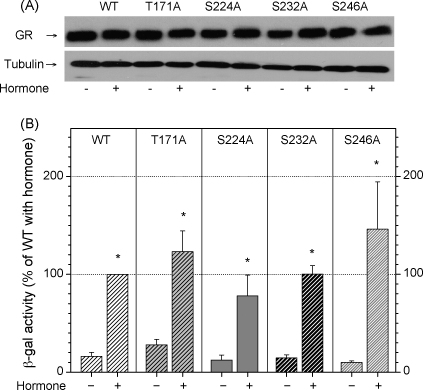
Effects of glucocorticoid receptor phosphorylation analysed in the constitutive overexpression system. (A) Western blot analysis of the expression levels of the WT and mutant GR derivatives expressed from the PG-1 plasmid. (B) Transcriptional activity of GR and its derivatives measured by β-galactosidase assay in the presence (+) or absence (−) of hormone. Results are presented as mean ± SEM (*n* = 6) from six or more independent measurements. Statistically significant differences between untreated samples and samples treated with hormone are given as *p < 0.05.

We next assessed whether the mutation of any of the phosphorylation sites on the receptor affects its transcriptional regulatory activities. We examined two expression systems in yeast cells: constitutive and regulated. Constitutive overexpression system uses PG1 plasmid carrying the cDNA for the wild type GR and phosphorylation site mutants (T171A, S224A, S232A and S246A) introduced into the yeast cells. The expression of the receptor was under the control of GPDH promoter that yields high and constitutive levels of protein expression. In addition, PG1 plasmid is of 2 μ origin conferring a high copy of the glucocorticoid receptor (10–40 copies per cell) and these factors in combination resulted in overexpression of the glucocorticoid receptor using this method. The wild type and the GR mutants were expressed in yeast and protein expression levels assessed by western blot analysis of cellular extracts (Fig. 4A). We observed that all GR derivatives were expressed at similar levels as compared to tubulin protein levels that served as the loading control.

The transcriptional activity of GR was analysed using a reporter gene containing TAT3 derived GREs fused to β-galactosidase cDNA [5]. Both wild type and mutant GR transcriptional activity was induced by hormone addition (Fig. 4B) and this induction was statistically significant in all GR derivatives studied. Although there was a trend indicating impaired activity of the S224A and S232A receptor mutants and increased S246A receptor activity in the presence of hormone as compared to the wild type GR (Fig. 4B), these differences were not statistically significant. Therefore, these effects were modest and unsuitable for the genetic screening intending to isolate proteins that display differential binding patterns depending on the GR phosphorylation status.

### 3.4 Effects of phosphorylation site mutations on receptor activity—regulable expression system

**Fig. 5:**
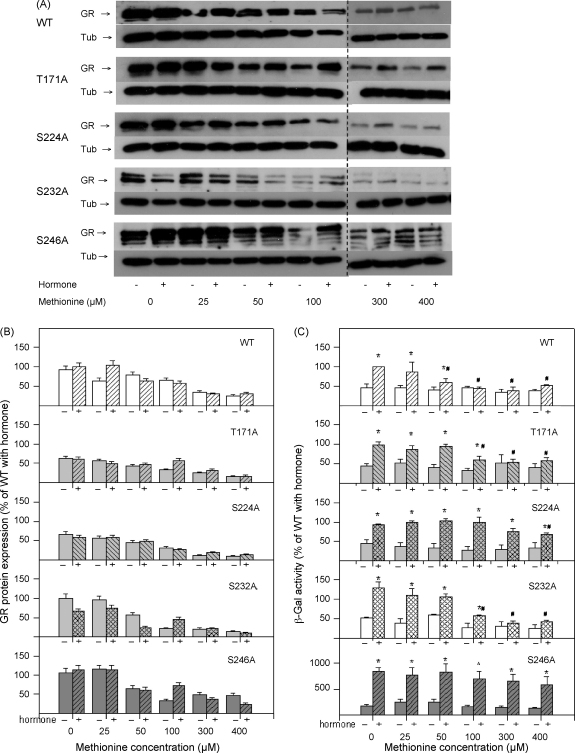
Effect of glucocorticoid receptor phosphorylation analysed in the regulable expression system. (A) Western blot analysis of the expression levels of the WT and mutant GR derivatives expressed from the p414 MET25 plasmid. (B) Quantification of the WT and mutant GR protein levels. (C) Transcriptional activity of the wild type GR or its derivatives was measured using β-galactosidase assay with the increasing concentration of methionine in the absence and presence of hormone as indicated. Results are presented as mean ± SEM (*n* = 6) from six or more independent measurements. Statistically significant differences between untreated samples and samples treated with hormone are given as *p < 0.05. Statistically significant differences between sample treated with hormone in the absence of methionine (met 0), and samples treated with hormone in the presence of methionine (25–400 μM met), are given as ^#^*p* < 0.05.

To establish an experimental system where the expression and cellular concentration of the GR can be rigorously controlled, we subcloned the cDNA of the wild type GR and GR carrying phosphorylation site mutations cDNA into the p414 MET25 vector. This vector is a pRS vector carrying methionine promoter and can express one copy per cell, which is lower than the PG1 vector driven genes. The expression of genes driven by the p414 MET25 promoter can be regulated by the amount of methionine in the media [23]. In the absence of methionine, this promoter is expressed at the highest level whereas concentration dependent levels of GR protein can be achieved by increasing methionine concentrations. The expression of the GR protein in this system shown in Fig. 5A and B indicates that the wild type and mutant receptors were expressed at similar levels and that the GR protein levels decreased with the increasing methionine concentrations. In the next series of experiments we tested the effects of phosphorylation site mutations on the GR transcriptional activity in the presence of increasing concentrations of methionine.

The activity of the wild type receptor increased with hormone addition and this increase was statistically significant up to methionine concentration of 50 μM (Fig. 5C, first panel). At methionine concentrations up to 100 μM the T171A and S232A GR mutants exhibited hormone induced transcription whereas higher methionine concentrations rendered these derivatives transcriptionally inactive. In contrast, activity of S224A and S246A GR derivatives was hormone dependent across all methionine concentration (Fig. 5C). In hormone-treated cells, WT, T171A and S232A receptors demonstrated declining transcriptional activity that was dependent on increasing methionine concentrations whereas S224A and S246A were mostly unaffected by methionine levels (Fig. 5C). The mutation of S246A clearly shows that this receptor derivative is a more potent activator of transcription than the wild type receptor suggesting that the phosphorylation of this amino acid has inhibitory effect on receptor function. Increased activity of this mutant was observed in both the absence and the presence of hormone.

## 4 Discussion

In this study we examined the phosphorylation of the glucocorticoid receptor (GR) expressed in yeast *S. cerevisiae*. Ectopically expressed receptor is competent for transcriptional regulation and signal transduction despite the fact that no endogenous glucocorticoid receptor has been detected in yeast [5,13]. Orthophosphate labeling *in vivo* and V8 protease digestion that yields simple and clear phosphopeptide pattern indicated that GR phosphorylation is similar in yeast and mammalian cells (Fig. 1). The obtained maps were substantially simpler in all cases, than the phosphotryptic maps we previously described [14], reflecting the fact that there were fewer primary cleavage sites for this protease, as well as fewer partial products generated by incomplete digestion. Using this method, we find that the mutation of S246 into alanine increases GR phosphorylation on S224 and S232 (Fig. 2). This conclusion was supported by the use of synthetic nonradioactive phosphopeptides that recapitulated *in vivo* peptide pattern. In addition, there were no differences in solubility of these synthetic phosphopeptides supporting the notion that interdependence of phosphorylation sites is conserved in yeast and mammalian cells suggesting that yeast homologues of cofactors [15,18] potentially involved in conferring this interdependence are conserved in yeast. Substantial body of evidence has indicated that phosphorylation is a very important post-translational modification regulating the function of many transcription factors. As such phosphorylation has been studied extensively in steroid receptors (SR) and the phosphorylated residues within GR, progesterone and oestrogen receptors have been determined in human, mouse, rat, chicken and yeast cells [2,16,17]. These residues are mainly localized within the N-terminal transcriptional-activating domain of the receptor (AF1) implying that phosphorylation is a potential regulator of GR transcriptional activity. Overexpression of the wild type and the above mentioned mutant GR proteins in yeast indicated that differences in the transcriptional potency of these derivatives were not statistically significant, although potential inhibitory trend of the MAPK and stimulatory tendency of the CDK targeted sites were observed, possibly due to GR overexpression masking the effects of GR phosphorylation (Fig. 4). In agreement with our results presented in Fig. 4, it has been shown that the mutation of some phosphorylation sites of mouse GR expressed in yeast or mammalian cells have modest or no effect on receptor transcriptional function when receptor was constitutively overexpressed [26–28]. However, recent reports have described transient and target gene specific effects of kinase activation on endogenous GR transcriptional activity [10,11].

The above mentioned difficulties in studying the effects of phosphorylation on GR function justified the effort to develop a controllable and genetically tractable system to further investigate this important post-translational modification. For this purpose we generated series of constructs carrying promoter which is regulated by methionine concentration expressing wild type and GR derivatives bearing mutations in four phosphorylation sites. Our results indicate that the concentration of the receptor is one of the crucial factors determining its transcriptional activity and different mutants display varying degree of dosage dependency (Fig. 5). For example, S246A mutant shows increased activity compared to the wild type receptor throughout the range of methionine concentrations and its activity is largely independent on the receptor dosage. S224A mutant activity is mainly independent from the GR protein levels. However, the wild type, T171A and S232A receptors display decreased activity with decreased levels of the receptor protein. These results suggest that there is a link between GR phosphorylation and its protein stability [8] as reported for other SRs [29]. Perhaps phosphorylation state controls GR ubiquitination and/or recruitment of components of proteasome and this process is linked to GR transcriptional activity [29,30]. We have observed increased degradation of the S246A derivative (Fig. 5A) indicating potential involvement of phosphorylation in control of the GR protein levels, but further investigation is needed to confirm this conclusion. Phosphorylation of S246 by JNK has been shown to increase GR sumoylation and mixed SUMO and ubiquitin chains have been proposed to play a role in transcriptional regulation and protein stability [10]. In fact, the link between proteasome and transcription has been established for several other transcription factors [31]. Together, our results support a model in which phosphorylation of GR at S246 compromises GR transcriptional activity by preventing phosphorylation at S224 and S232 (Fig. 3 and [15]). Possible molecular mechanisms include alternative protein–protein interactions between differentially phosphorylated GR and its transcriptional cofactors. Similar mechanism has been described for the interaction between CREB transcription factor and its coactivator protein p300/CBP [16,17,32,33,34]. Recent report by Chen et al. indicates that S211 (a human analogue of the rat S232) phosphorylation regulates cofactor interaction in a target gene specific way and that effects of phosphorylation are dependent on the amount of activated receptor [35]. Although experimental approach described here is limited to a single target gene, it offers the advantage of developing genetic screen for protein–protein interactions that are dependent on the GR dosage and on the GR phosphorylation. We speculate that GR protein levels may be important in the determination of the GR phosphorylation effects [8,27] which may be of special importance *in vivo* under the conditions of low endogenous hormone levels [36]. Differential interaction of the hyper- or hypo-phosphorylated receptor with components of the proteasome might affect its half-life and ensure that GR activity and its stability are regulated in coordinated fashion.

In conclusion, the simple system described in this report will be useful in the investigation of the complex effects of phosphorylation by providing detailed insight in several aspects of the physiological role of phosphorylation of steroid receptors and other transcription factors and it is the initial step towards isolation of cofactors which demonstrate differential interaction profile with the hyper versus the hypo-phosphorylated GR.
